# Utilising the ‘Getting to Outcomes^®^’ Framework in Community Engagement for Development and Implementation of Sickle Cell Disease Newborn Screening in Kaduna State, Nigeria

**DOI:** 10.3390/ijns4040033

**Published:** 2018-11-16

**Authors:** Baba P.D. Inusa, Kofi A. Anie, Andrea Lamont, Livingstone G. Dogara, Bola Ojo, Ifeoma Ijei, Wale Atoyebi, Larai Gwani, Esther Gani, Lewis Hsu

**Affiliations:** 1 Department Evelina Children’s Hospital, Guy’s and St Thomas’ NHS Foundation Trust, London SE1 7EH, UK; 2 Department of Haematology and Sickle Cell Centre, London North West University Healthcare NHS Trust and Imperial College, London NW10 7NS, UK; 3 Department of Implementation Science, University of South Carolina, Columbia, SC 29208, USA; 4 Department of Haematology, School of Medicine , Kaduna State University, Barau Dikko Teaching Hospital, Kaduna 800212, Nigeria; 5 Sickle Cell Cohort Research Foundation, WUSE Zone II, Abuja 70032, Nigeria; 6 Department of Haematology, Oxford University Hospitals NHS Foundation Trust, Oxford OX3 9DU, UK; 7 Kaduna State Assembly Office, Kaduna 800212, Nigeria; 8 Library Department, Kaduna State University, Kaduna 800241, Nigeria; 9 Department of Pediatric Hematology-Oncology, University of Illinois at Chicago, Chicago, IL 60612, USA

**Keywords:** Sickle Cell Disease, ‘Getting to Outcomes’, newborn screening), sub-Saharan Africa, Nigeria, Kaduna State, implementation science, public health engagement

## Abstract

Background: Sickle Cell Disease (SCD) has been designated by WHO as a public health problem in sub-Saharan Africa, and the development of newborn screening (NBS) is crucial to the reduction of high SCD morbidity and mortality. Strategies from the field of implementation science can be useful for supporting the translation of NBS evidence from high income countries to the unique cultural context of sub-Saharan Africa. One such strategy is community engagement at all levels of the healthcare system, and a widely-used implementation science framework, “Getting to Outcomes^®^” (GTO), which incorporates continuous multilevel evaluation by stakeholders about the quality of the implementation. Objectives: (1) to obtain critical information on potential barriers to NBS in the disparate ethnic groups and settings (rural and urban) in the healthcare system of Kaduna State in Nigeria; and, (2) to assist in the readiness assessment of Kaduna in the implementation of a sustainable NBS programme for SCD. Methods: Needs assessment was conducted with stakeholder focus groups for two days in Kaduna state, Nigeria, in November 2017. Results: The two-day focus group workshop had a total of 52 participants. Asking and answering the 10 GTO accountability questions provided a structured format to understand strengths and weaknesses in implementation. For example, we found a major communication gap between policy-makers and user groups. Conclusion: In a two-day community engagement workshop, stakeholders worked successfully together to address SCD issues, to engage with each other, to share knowledge, and to prepare to build NBS for SCD in the existing healthcare system.

## 1. Introduction

Sickle Cell Disease (SCD) has been designated by the World Health Organisation (WHO) as a public health problem in sub-Saharan Africa [1,2,3]. It is projected that, unless specific action is taken, the burden of disease will continue to increase into 2050, especially in Nigeria and Democratic Republic of Congo, where this increase is estimated to be more than 100% [4]. The number of annual births with SCD is estimated to be 100,000 to 150,000 in Nigeria. Our pilot Newborn screening (NBS) study of infants up to six months old in an area within Kaduna State, Nigeria, reported an incidence of 1.7% [4,5], which suggests that over 4000 babies with SCD are born every year (based on 240,000 annual overall births per state). Consistent with WHO’s call to action, national and regional policies for the management and control of SCD are required, especially in the view of limited resources across most of sub-Saharan Africa. SCD represents an urgent health burden, both in terms of mortality and morbidity. It is estimated that it accounts for 8–16% of under-five mortality in sub-Saharan Africa [6]. Mortality among children with SCD in Africa is estimated at 50% to 90% by 10 years of age, mostly from preventable infections [2].

Effective management of SCD should incorporate NBS with the prevention of infections (including pneumococcal septicaemia and malaria), parental education, and support at all levels of healthcare provision to enable the timely recognition of SCD complications and health maintenance. The development of NBS programmes in sub-Saharan Africa is crucial to the reduction of high infant mortality. These programmes must be guided by empirical evidence, often accumulated in high income countries, such as United States of America (USA) and United Kingdom (UK), and simultaneously fit within the unique cultural context of sub-Saharan Africa (which is very distinct from the setting of the original clinical trial). This often poses a challenge in implementation where the original trial does not fit with the local context. Strategies from the field of implementation science, defined as the “scientific study of methods to promote the systematic uptake of research findings and other evidence-based practices into routine practice, and, hence, to improve the quality and effectiveness of health services and care” [7] can be useful for supporting the translation of evidence from clinical trials to implementation in contexts vastly different from that originally employed in the clinical trial, such as Kaduna State in Africa. One such strategy is community engagement at all levels of the healthcare system [8].

Kaduna State in northern Nigeria was the country’s old colonial capital, it is a microcosm of the entire country, and has a population of over eight million made up of over 60 different ethnic groups, with 23 local governments, three geopolitical (senatorial) zones, with over 30 health care facilities for secondary care, two academic institutions (tertiary care and undergraduate training), and five teaching hospitals (tertiary care) hospitals. The academic institutions are Ahmadu Bello University Teaching Hospital and Barau Dikko Teaching Hospital, Kaduna State University, and the other four tertiary care level hospitals are National Eye Centre, National Ear Care Centre, Federal Neuro-Psychiatric Hospital, and 44 Nigerian Armed Forces Reference Hospital. The State offers free healthcare for pregnant women and children up to five years of age. Kaduna State Primary Health Care Agency is led by an Executive Secretary to oversee primary care centres and clinics in conjunction with the local governments.

We embarked on community engagement as the initial step to informing the development and implementation of an NBS programme for SCD in Kaduna State. Community engagement has been broadly defined as involving communities in information giving, consultation, decision-making, planning, co-design, governance, and delivery of services [9]. This was an early phase of sustained engagement with a broad range of community representatives to be inclusive and aimed for equal partnership. Furthermore, the application of implementation science within health systems is of benefit to the development and implementation of health interventions. Despite favourable evidence in clinical trials, programmes often fail to reach their desired outcomes in the real world due to limitations outside the trial environment and challenges with implementation.

Implementation science provides strategies to help guide implementation, therefore improving access to evidence-based services that fit with the culture of the population in need. The first phase usually consists of descriptive, formative research to better understand the major implementation challenges and to design potential strategies to overcome these [10]. We employed a widely-used implementation science framework, “Getting to Outcomes^®^” (GTO), which incorporates continuous multilevel evaluation by stakeholders about the quality of the implementation [11,12,13]. GTO is a 10-step system of accountability that guides the user through the process of planning, monitoring, and evaluating programmes. The continuous evaluation facilitates adaptation of the programme to local capacity and motivation for change, which maximizes the chances of programme success.

The objectives of the community engagement were two-fold. First, to obtain critical information pertaining to disparate ethnic groups and settings (rural and urban), including potential barriers to a successful NBS with the Kaduna State healthcare system and subsequent policy implementation. Second, to assist in the readiness assessment of Kaduna State in the implementation of a sustainable NBS programme for SCD.

## 2. Methods

Qualitative research methodology was employed in a two-day focus group workshop with an identical format over the two days in Kaduna. A representative group of participants were invited for each of the two focus group sessions. These comprised parents of children with SCD, adults with SCD, representatives of patient association and support groups, community leaders, health professionals, and policy-makers from the three health zones in Kaduna state, including nurses and midwives. Community health extensions workers from primary healthcare centres, doctors, and nurses from general and teaching hospitals were among the participants. In addition, five participants from the neighbouring Niger State were invited to the first focus group session to highlight the anticipated differences between states.

Focus group discussions were facilitated by a faculty of five international and local experts in SCD from the UK and Nigeria, including paediatricians, haematologists, a psychologist, and a professional in community engagement. The focus group format included brief introductory lectures on SCD and NBS. This was followed by a series of 10 questions based on the ten-point GTO framework for discussion. Each participant was given the opportunity and was encouraged to be candid with their responses and discussions in a relaxed and open atmosphere and speak in any language of their preference. Proceedings were transcribed by a professional scribe and audio-recorded. Subsequently, transcripts were produced from the audio recordings by two professionals that were experienced in transcribing (LG) and qualitative research (EG). Their combined report was reviewed by the facilitators of focus groups for accuracy and consistency (KAA, BO, and BI).

## 3. Results

There was a total of 52 participants for the two-day focus group workshop (Table 1). Discussions based on the GTO questions and additional issues are summarised by themes generated below.

### 3.1. Objectives of a SCD Programme

- Early detection and reduction of SCD in our communities
- To offer subsidised testing and treatment
- To minimize the cost of treatment and maintenance
- Reduce psychological and emotional trauma amongst family members
- To reduce the financial drain on the families of SCD patients
- Increase awareness of SCD most especially in the rural areas
- Improve the health status of SCD patients
- Eradicate stigma
- Healthy communities to function better
- Accurate data to inform policy makers in improved planning
- Give hope to patients with SCD to live normal fulfilled lives
- Improve standard of diagnosis to rule out confusion
- Increase the life expectancy of patients and eradication of SCD
- Reduce morbidity and mortality

### 3.2. Perceptions about NBS

- Early diagnosis and administering Penicillin improve on the patient’s life expectancy
- Strong perception about SCD not having a cure affects the minds of families
- Poverty and financial constraint hinder families from accessing NBS
- Myths and traditional beliefs about SCD being associated with witchcraft creates an obstacle to NBS
- Most SCD babies not tested at birth end up dying from malaria even before SCD is detected

### 3.3. Implementation of NBS

- The early diagnosis should be at primary, secondary and tertiary health care centres
- Parents of affected children should be confidentially informed of the implication of SCD and how to prepare for the child’s welfare
- World Sickle Cell Day should be emphasised with adequate publicity
- Screening, diagnosis, counselling and service delivery should be inter faced
- Blood samples should be taken at birth and in post-natal clinics
- Incentivising the process by giving out souvenirs
- NBS should be free and patients be given free or subsidized medication
- The Government should give SCD a priority

### 3.4. Why We Need a NBS Programme

- To create the opportunity for effective management of SCD
- To inform the community on the importance of screening
- To inform parents on how to prepare for the child’s welfare
- Early detection will make the government have up to date data on SCD for adequate planning
- To increase the chances of controlling the disease
- To help in reducing stigma and disabuse the perception of the community
- To properly manage patients and parents

### 3.5. Best Practices to Adopt

- Community based approach by involving Volunteer Community Mobilisers (VCMs) and Traditional Birth Attendants (TBAs)
- Facility based approach
- Utilising media to disseminate information through drama on radio and television
- Incorporate the importance of NBS during antenatal health talks
- Involve community and religious organizations for sensitization campaigns like in the case of the “child spacing” campaigns
- Development partners, NGOs and media collaboration to expand
- More Sickle cell centres should be made available, accessible and affordable
- Social networks should be utilized for campaigns of SCD
- Train existing staff and employ additional qualified staff to run the centres
- Compulsory routine testing at birth
- Build linkages between the community and health care facilities

### 3.6. Resources and Capacity Building Needed

- Train TBAs to use simple testing for NBS
- Train Village Community Mobilisers
- Train existing staff and employ additional qualified professionals
- Existing health facilities should be equipped
- Build on existing HIV infrastructure
- Technical and financial support from development partners, and charitable organizations
- Continuous advocacy for dissemination of the facts about SCD
- Newborn testing should be available, accessible and affordable

### 3.7. How to Evaluate the Success of the Programme

- Using existing data to plan
- Correct and appropriate documentation is essential for evaluation
- Continuous monitoring of the programme
- Training and re-training of personnel

In addition, core themes identified within the GTO framework and categorised by the type of participant or institution are presented in Table 2.

## 4. Discussion

Readiness is part of GTO, but what we did in this focus group was broader than readiness alone. We organized the findings by GTO steps, which served to (1) understand differences in perspectives across the different levels (this is important for addressing potential barriers) and (2) to remain accountable for implementation. From an implementation standpoint, one of the challenges faced in health care settings is the transport of interventions from a research trial to naturalistic setting. There are many factors that get in the way of successful implementation in naturalistic settings, especially in complex settings, like the multilevel healthcare structure in Kaduna. Differences in the vision, needs, resources, and goals of different levels of the health system may get in the way of successful implementation. This complexity is compounded by differences in contextual factors between the setting of the original clinical trial of the intervention and the local context where the intervention is being implemented. Most likely adaptations are needed to achieve the similar outcomes of a well-funded clinical trial in a developing country. In order to identify which adaptations are needed, and at which level these adaptations are needed, community engagement at each healthcare level is needed.

SCD poses a major public health problem in Nigeria. Community engagement as a first step to developing and implementing a sustainable NBS programme was carried out by SCD experts from UK, USA, and Nigeria, working with a charity in Nigeria called the Sickle Cell Cohort Research (SCORE) Foundation. Focus group discussions employing an implementation science approach with patients, parents, community leaders, doctors, nurses, and community health workers allowed active participation and important information to be gathered about the difficulties and solutions for testing newborn babies in these communities, including cultural and religious beliefs.

This study employed a well-known implementation framework to guide community engagement. Through focus groups, we uncovered certain areas where potential barriers to implementation may exist and where certain adaptations may be needed to improve the chances of achieving programmatic success. For example, we found a major communication gap between policy-makers and user groups. There is an absence of patient-users consultation within the state policy framework and therefore the lack of opportunity to incorporate their views in service planning and implementation. Asking and answering the 10 GTO accountability questions provided a structured format to understand the strengths and weaknesses in the implementation setting. This led nicely to the development of plans that support quality implementation. In this way, the hospitals will be more prepared for implementation and increase their chances of programmatic success.

The goals and objectives were addressed. Outcomes include the opportunity for participants working together to address SCD issues, to network, and engage with each other. Shared knowledge by participants, greater awareness of what is in place albeit on a small scale. Some myths and misinformation were addressed. There is no doubt that the importance of NBS for SCD programme development and implementation in Kaduna State, Niger State and the entire country cannot be over emphasised. To ensure the sustainability of the programme, the government has to be fully committed to it by providing the legal framework, policies, and adequate funding. It is also important to note that issues such as lack of public awareness and concerns could be barriers to a successful programme. Therefore, it is necessary to educate the general public through media campaigns, and advocate in partnership with the support of religious and traditional leaders within.

## 5. Summary and Conclusions

The two-day workshop successfully set the stage for the development and implementation plan of the NBS programme for SCD communities. Recommendations for the next steps to developing a Kaduna State NBS for SCD programme were made to the State’s Commissioner of Health, and subsequently an initial four-day training workshop was organised prior to step by step implementation: (i) Procurement of reagents (ii) collection of blood spots from one local government area (1/23) of the state to test robustness of specimen collection, transportation to the laboratory, analysis turnaround time; result disclosure to families, (iii) counselling to families; and, (iv) referral to treatment clinic. A number of key themes from this ‘Getting To Outcomes’ (10 steps) assessment process require urgent implementation by Kaduna State through the setting up of steering committee to address the issues that were raised regarding the Objectives of a SCD programme, Perceptions, and Implementation of NBS. For the State to adopt Community based approach by involving Volunteer Community Mobilisers (VCMs) and Traditional Birth Attendants (TBAs) for maximum benefit and to ensure that a robust monitoring and evaluation process is in place.

## Figures and Tables

**Table 1:** Participants of the Two-Day Community Engagement Focus Group Sessions.

Institution or Participant	Number
Adult with Sickle Cell Disease	2
Ahmadu Bello University Teaching Hospital–Zaria	4
Ahmadu Bello University Teaching Hospital School of Nursing–Zaria	1
Barau Dikko Teaching Hospital–Kaduna	8
Fantsuam Foundation–Kafanchan	3
Gambo Sawaba Memorial Hospital–Zaria	1
Federal Ministry of Finance–Abuja (Independent Participant)	1
Kaduna State Primary Healthcare Development Agency	2
Media Representatives	2
Mil-Goma Community Leaders–Zazzau Emirate	2
Niger State Government–(Jumai Babangida Aliyu Maternal and Neonatal Hospital) Minna, Niger State	5
Panaf Schools–Kaduna	2
Parent of a Child/Children with Sickle Cell Disease	3
Rahma Integrated Sickle Cell Research Centre–Kaduna	1
Safiya Sickle Cell Foundation Zaria–Kaduna and Abuja	3
Samira Sanusi Sickle Cell Foundation–Kaduna	4
Sickle Cell Health Promotion Centre–Kaduna	2
Sir Patrick Ibrahim Yakowa Hospital–Kafanchan	4
Kaduna State House of Assembly	1
Kaduna State Ministry of Health and Human Services	1

**Table 2:** Ten Steps of the “Getting to Outcomes^®^ Framework for Sickle Cell Disease New Born Screening and Key Messages from Participants.

	Parent of Sickle Cell Disease (SCD) Child	Community Health Worker	Health Centre Doctor	Health System Hospital Administrator	Laboratory Technician	Patient Organisation Representative
**Step 1: Needs & Resources**	Early diagnosis & pre-marital counselling	Early awareness of SCD status	Early diagnosis & lack of treatment facilities	Innovative utilisation of resources	Equipment, reagents & quality assurance	Use of media for public awareness
**Step 2: Goals & Objectives**	Knowledge of diagnosis and access to treatment	Address ignorance, stigma & beliefs	Early detection of SCD and provision of medical care	Equity on service provision for SCD similar to HIV	To eliminate errors in diagnosis	Public perception about SCD
**Step 3: Best Practices**	Immunisation programme which is accessible	Strong educational elements of family planning campaign	HIV/AIDS programme structure & funding	Low cost intervention that is affordable	Reduce false positives & false negatives results	SCD education for families & general public
**Step 4: Programme Fit for NBS**	Testing during other clinics such as immunisation	Community worker leadership important	Primary health care system to reach local communities	Combine with other dried blood sample testing	Staff trained for IEF ^a^ & would like skills in HPLC ^b^ in addition	Encourage community participation
**Step 5: Capacity for NBS**	Staff must be competent	Partnership with community	Shortages of staff, medicines & development of skills	Limited resources, 3 tiers of government & community participation	Reagents supply, storage & inventory	Public engagement and sensitisation
**Step 6: NBS Implementation Plan**	Provide medicines & access to staff	Counselling, treatment for patients & families	Health status, treatment, tracking & follow up	Need to know SCD burden, resource implication	Clear standard operating procedures	Address myths & stigma
**Step 7: Evaluation for NBS**	Is my baby growing well?	Reporting outcome of babies visiting the SCD centre, verbal autopsies	Diagnosed babies receiving penicillin & attending SCD clinic	Infant & childhood mortality, immunisation coverage	Monthly & quarterly arranged Quality Assurance	Parliamentary oversight & reports to constituents.
**Step 8: NBS Outcome Evaluation**	Knowledgeable staff & a Sickle Cell Centre	Number of patients accessing counselling services	Percentage of diagnosed babies with SCD, penicillin prophylaxis	Survival for SCD children at 1, 5 &10 years of age	Accurate & timeliness of laboratory results	A sickle cell centre for Kaduna state
**Step 9: Continuous Quality Improvement**	Parent support & input in care	Education & step-down training	Teleconference discussion on NBS programme results & troubleshooting	Continuous assessment & Peer Review Systems	Weekly quality reports on results, timeliness & errors	Sensitise general public, religious & community leaders
**Step 10: Sustainability of NBS Programme**	Not limited to a state governor’s term in office	Involve all sectors of health care	Multidisciplinary team, government support	Involvement of all parties	Train personnel for additional laboratory procedures	Educate to accept responsibility of both men & women

^a^ Isoelectric Focusing (IEF). ^b^ High Performance Liquid Chromatography (HPLC).
